# Information and Communication Technology Changes in Long-Term Care Due to COVID-19

**DOI:** 10.1093/geroni/igab046.840

**Published:** 2021-12-17

**Authors:** Amy Schuster, Shelia Cotten

**Affiliations:** Clemson University, Clemson, South Carolina, United States

## Abstract

Residents of long-term care (LTC) facilities (e.g., nursing homes, assisted living facilities) have historically been vulnerable to feelings of loneliness and social isolation. Due to the COVID-19 pandemic, LTC facilities were required to restrict public access in March 2020. LTC communities were not prepared for the residents’ increased socioemotional needs which arose because of the mandated facility lockdown. This study investigated ICT use in LTC facilities and how ICTs are being used by residents since the onset of the COVID19 pandemic. Seventy LTC administrators in South Carolina (12 nursing homes and 58 assisted living facilities) completed an online survey exploring ICT access and use in LTC facilities and whether access and use changed as a result of COVID-19. Administrators from fifty-three percent of LTC facilities reported purchasing ICTs for their residents to use for communicating with family members and telehealth since the onset of COVID-19. LTC administrators reported that using the ICTs helped residents to socialize more frequently and feel more socially connected to their family members, friends and/or other residents. Barriers to ICT use included staff not having time to assist residents with technology, broken technology, and residents not wanting to share technology. LTC facilities were not adequately prepared to support the socioemotional needs of their residents in the event of a federally mandated facility lockdown. Future research should investigate the ICTs available for residents’ use in a national sample of LTC facilities and how LTC administrators adapted the ICTs available as a result of their experiences with COVID-19.

